# Phacoemulsification Techniques and Their Effects on Corneal Endothelial Cells and Visual Acuity: A Review of "Direct-Chop" and "Stop-and-Chop" Approaches Under Topical Anesthesia

**DOI:** 10.7759/cureus.66587

**Published:** 2024-08-10

**Authors:** Devwrath Upasani, Sachin Daigavane

**Affiliations:** 1 Ophthalmology, Jawaharlal Nehru Medical College, Datta Meghe Institute of Higher Education and Research, Wardha, IND

**Keywords:** direct-chop technique, stop-and-chop technique, topical anesthesia, visual acuity, corneal endothelial cells, phacoemulsification

## Abstract

Phacoemulsification is a widely adopted technique in cataract surgery that offers a minimally invasive approach to lens removal and intraocular lens implantation. Among the various methods of phacoemulsification, "direct-chop" and "stop-and-chop" techniques are particularly notable for their efficiency and safety profiles. This review aims to evaluate the effects of these two techniques on corneal endothelial cells and visual acuity, specifically under topical anesthesia. Cataract surgery outcomes hinge on the preservation of corneal endothelial cells and the achievement of optimal visual acuity. Endothelial cell loss can lead to corneal decompensation, while visual acuity is a primary measure of surgical success. The "direct-chop" technique involves the immediate chopping of the lens nucleus after groove creation, reducing phacoemulsification time and energy. Conversely, the "stop-and-chop" technique incorporates a central groove before chopping, offering increased control and safety. This review synthesizes current research and clinical studies to compare these techniques, focusing on their respective impacts on endothelial cell count and postoperative visual acuity. It examines the advantages and disadvantages of each approach, considers the role of surgeon experience, phacoemulsification energy, and anterior chamber stability, and assesses patient outcomes under topical anesthesia. The findings aim to provide insights that can guide surgeons in selecting the most appropriate technique for their patients, ultimately enhancing surgical outcomes by ensuring the preservation of corneal health and the achievement of superior visual acuity.

## Introduction and background

Cataract surgery is one of the most commonly performed surgical procedures worldwide, primarily aimed at restoring vision impaired by the clouding of the eye's natural lens [1]. The advent of phacoemulsification has revolutionized cataract surgery by offering a minimally invasive technique that emulsifies the opaque lens using ultrasonic vibrations, allowing for its removal through a small incision. This method not only reduces recovery time but also minimizes complications compared to older techniques such as extracapsular cataract extraction (ECCE) [1]. Phacoemulsification involves the use of a phaco probe, which delivers ultrasonic energy to break up the lens into tiny fragments that can be aspirated out of the eye. This procedure is typically followed by the implantation of an intraocular lens (IOL) to replace the removed lens, thus restoring the patient's vision. Over the years, various techniques within phacoemulsification have been developed to optimize outcomes, including the "direct-chop" and "stop-and-chop" approaches [2].

The preservation of corneal endothelial cells (CECs) is crucial in cataract surgery as these cells play a vital role in maintaining corneal transparency by regulating fluid and solute transport across the cornea. Endothelial cell loss during phacoemulsification can lead to corneal edema and decompensation, resulting in compromised vision and the potential need for further surgical intervention such as corneal transplantation [3]. Maintaining visual acuity is the primary goal of cataract surgery, and successful outcomes depend not only on the effective removal of the cataract but also on minimizing intraoperative and postoperative complications. The choice of phacoemulsification technique can significantly impact both the preservation of endothelial cells and the final visual acuity, making it essential to evaluate the benefits and drawbacks of different approaches [4].

The "direct-chop" technique, introduced by Nagahara, involves the use of a chopper to split the nucleus of the lens directly after initial groove creation. This method is known for its efficiency in reducing phacoemulsification time and energy, potentially leading to better preservation of CECs [5]. On the other hand, the "stop-and-chop" technique, popularized by Paul Koch, combines elements of both divide-and-conquer and chop methods. It starts with the creation of a central groove ("stop"), followed by the chopping of the nucleus into smaller pieces. This technique is designed to offer greater control and safety, especially for less experienced surgeons [6]. This review aims to compare the "direct-chop" and "stop-and-chop" techniques in terms of their effects on CECs and visual acuity when performed under topical anesthesia. By examining current research and clinical studies, this review seeks to provide insights into the advantages and disadvantages of each technique, thereby guiding surgeons in their choice of the most appropriate method for their patients. Ultimately, the goal is to optimize surgical outcomes by ensuring both the preservation of corneal health and the achievement of optimal visual acuity.

## Review

Phacoemulsification techniques

Overview of Phacoemulsification

Phacoemulsification is a widely utilized surgical technique for cataract removal, renowned for its efficiency and minimal invasiveness. This procedure employs ultrasonic energy to emulsify the eye's cloudy lens, which is typically caused by cataracts. The process begins with the insertion of an ultrasonic probe through a small incision in the eye. This probe emits high-frequency sound waves (usually between 27 kHz and 60 kHz) that break the lens into tiny fragments and then aspirate from the eye. A balanced salt solution maintains the anterior chamber and prevents collapse during the procedure [7]. The surgical technique for phacoemulsification involves several key steps. First, patients receive topical or local anesthesia to minimize discomfort. Preoperative assessments evaluate the cataract's hardness and the eye's overall health. A small incision, typically around 2.2-3.0 mm, is made in the cornea, allowing access to the lens without sutures [8]. Once the incision is made, the ultrasonic probe is inserted, and the lens is broken down into smaller pieces. Surgeons may employ "stop-and-chop" or "direct-chop" to facilitate emulsification. After the lens is emulsified, the fragments are suctioned out of the eye. Finally, a foldable IOL is inserted through the same incision, replacing the natural lens and restoring vision [9]. Phacoemulsification offers several advantages contributing to its status as the gold standard for cataract surgery. Its minimally invasive nature means that the small incision reduces healing time and minimizes the risk of astigmatism. Additionally, the procedure is typically performed on an outpatient basis, allowing most patients to return home shortly after surgery. Many individuals experience improved vision within days, and the overall success rate of phacoemulsification is high, particularly in uncomplicated cases [10]. Despite its many benefits, phacoemulsification is not without risks. Potential complications include infection, retinal detachment, glaucoma, and secondary cataracts. However, these complications are rare, especially with prophylactic antibiotics following surgery [11].

Description of the "Direct-Chop" Technique

The "direct-chop" technique in phacoemulsification is a critical cataract surgery method and is particularly effective in challenging cases. Begin by ensuring an adequately sized capsulorhexis and perform thorough hydrodissection and hydrodelineation to separate the nucleus from the capsule [12]. Use the phaco tip to engage the nucleus, embedding it deeply into the center. Employ a chopper (either horizontal or vertical) to create a fracture in the nucleus. In horizontal chopping, the chopper moves toward the phaco tip and then laterally to cleave the nucleus. In vertical chopping, the chopper moves downward next to the phaco tip to split the nucleus. After the initial chop, rotate the nucleus and repeat the chopping process to create smaller fragments, which can then be aspirated or emulsified. Continue fragmenting the nucleus until all pieces are removed from the capsular bag [13]. The direct-chop technique minimizes the amount of ultrasound energy used, decreasing the risk of thermal damage to surrounding tissues. It applies less force against the zonules than traditional methods, reducing the risk of zonular dialysis. The technique is beneficial in cases with small pupils, mature cataracts, or subluxated lenses, as it allows for effective nuclear disassembly without prolapsing the entire nucleus out of the capsular bag. Additionally, it offers better control during surgery, especially in dense nuclei, due to the manual forces applied [14]. Mastery of the direct-chop technique requires practice, and beginners may struggle with the initial chop, especially in harder cataracts. If the chopper is not positioned correctly or insufficient force is applied, the nucleus may not split completely, necessitating additional attempts. The technique is less effective on softer cataracts, where the chopper may not achieve a clean cut, leading to potential complications [15].

Description of the "Stop-and-Chop" Technique

The stop-and-chop technique is a hybrid phacoemulsification method that combines elements of the divide-and-conquer approach with the phaco-chop technique. This method is particularly beneficial for beginner surgeons due to its systematic steps and efficiency in nucleus division. The technique involves several key steps that ensure effective cataract removal while minimizing potential complications [16]. The procedure begins with careful preparation, including creating a sufficiently sized capsulorhexis of at least 5 mm diameter. Hydrodissection and hydrodelineation are then performed to facilitate the rotation of the nucleus [2]. The next step involves sculpting a longitudinal groove in the nucleus using moderate flow and low vacuum. This groove should be about one and a half phaco tips wide and three tips deep, allowing for a thin posterior plate that can be easily cracked. Once the groove is established, the surgeon cracks the nucleus into two hemisections by inserting the phaco tip and the chopper into the groove and applying pressure to divide the posterior plate [2]. After the nucleus is cracked, the hemisections are rotated 90 degrees to lie at the 6 and 12 o'clock positions. The settings are then adjusted to high vacuum and flow, allowing the surgeon to chop the hemisections into smaller pieces as needed, depending on the density of the cataract. Following this, the surgeon emulsifies the chopped pieces using the phaco tip while managing the second instrument to prevent occlusion. Finally, an irrigation/aspiration tip removes any remaining nuclear material, completing the procedure [6]. The stop-and-chop technique offers several advantages. It is particularly easy for novice surgeons to learn, as it simplifies the initial steps of nucleus division. Additionally, creating a groove before chopping minimizes the need for phaco power, reducing endothelial damage risk. The groove also allows for better access and manipulation of nuclear fragments, making removing them from the capsular bag easier than direct-chop techniques. Furthermore, it is a versatile stepping stone for surgeons transitioning to more advanced methods [6]. However, there are some disadvantages associated with the stop-and-chop technique. If not performed correctly, there can be risks such as posterior capsule rupture or incomplete nucleus division. The technique's effectiveness also relies on the surgeon's ability to create an adequate groove and perform the subsequent chopping. Additionally, while it is effective for many cataracts, the technique may not be as suitable for extremely dense nuclei, where more aggressive methods might be required [17].

Effects on CECs

Importance of CECs in Maintaining Corneal Transparency

**Figure 1:**
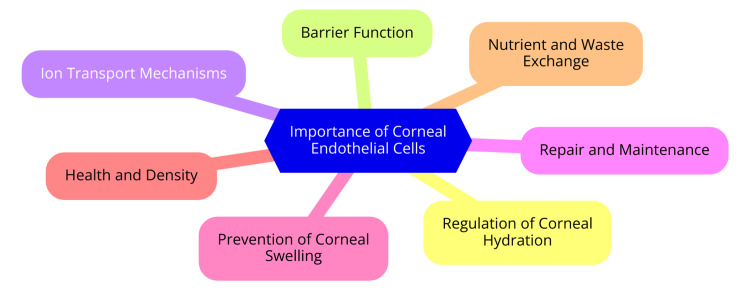
Importance of corneal endothelial cells in maintaining corneal transparency Image Credit: Dr. Devwrath Upasani

CECs are crucial in maintaining corneal transparency, essential for clear vision. These cells are responsible for two primary functions: providing a barrier and facilitating a pump mechanism that regulates corneal hydration [18]. The integrity of the corneal endothelium is vital for preventing excessive fluid accumulation in the cornea, as it acts as a selective barrier between the corneal stroma and the aqueous humor in the anterior chamber of the eye. The tight junctions formed between endothelial cells help maintain this barrier, regulating ions and nutrients while preventing the influx of unwanted substances [18]. In addition to their barrier function, CECs are integral to the cornea's pump function. This is primarily mediated by the Na+/K+-ATPase pump, which transports sodium ions from the corneal stroma and potassium ions into it. This process draws water out of the stroma, maintaining a relative dehydration essential for corneal clarity. The cornea must remain in a precise state of hydration to ensure light can pass through without distortion. When the pump function of CECs is compromised due to cell loss or dysfunction, it can lead to corneal edema, resulting in blurred vision and discomfort [19]. The cornea's transparency largely depends on the organization and hydration of the stroma, which is influenced by the health and functionality of the endothelial layer. When CECs are damaged or lost, the remaining cells can enlarge and migrate to cover the defects, but they cannot proliferate and replace the lost cells. This limited regenerative capacity means significant endothelial cell loss can lead to irreversible corneal decompensation and visual impairment [20]. Clinically, corneal endothelial dysfunction is a leading cause of corneal edema and visual impairment, often necessitating corneal transplantation. Given the global shortage of donor corneas, researchers are actively exploring alternatives, such as cultured endothelial cell therapies, to address this challenge. CECs maintain corneal transparency through their barrier and pump functions. Their health is crucial for preventing corneal edema and ensuring clear vision, highlighting the need to effectively manage conditions that affect these cells [21]. The importance of CECs in maintaining corneal transparency is shown in Figure 1.

Mechanisms of Endothelial Cell Damage During Phacoemulsification

Phacoemulsification, a widely used cataract surgery technique, can significantly damage CECs. Understanding the mechanisms of this damage is critical for improving surgical outcomes and preserving corneal health [22]. Ultrasound energy is one of the primary mechanisms of endothelial cell damage during phacoemulsification. This energy is essential for emulsifying the cataractous lens but can also cause mechanical and thermal injury to the corneal endothelium. Acoustic cavitation, a phenomenon induced by ultrasound waves, leads to the formation of bubbles in the aqueous humor that collapse violently. This collapse generates shock waves and turbulence, which can harm endothelial cells. Additionally, the collapse of these bubbles produces reactive oxygen species (ROS), contributing to oxidative stress and subsequent cell apoptosis [23]. Mechanical trauma is another significant factor in endothelial cell damage. This trauma occurs due to the direct contact of surgical instruments with the corneal endothelium. During surgery, fluid dynamics in the anterior chamber can create shear stress on the endothelial cells, leading to cell loss [24]. Moreover, contact between the IOL and the corneal endothelium can exacerbate this trauma, particularly if the IOL is not positioned correctly or there is excessive manipulation during the procedure. The interaction of nuclear fragments with the endothelium during lens fragmentation can further increase mechanical trauma, resulting in greater endothelial cell loss [24]. Thermal injury is also a concern during phacoemulsification. The ultrasound energy can generate heat, which may compromise the integrity and function of endothelial cells. Elevated temperatures can lead to cellular damage and contribute to the development of corneal edema after surgery. This thermal effect, combined with the mechanical and acoustic forces at play, underscores the complexity of the challenges faced during cataract surgery [25]. Finally, oxidative stress plays a crucial role in endothelial cell damage. The formation of ROS during phacoemulsification can induce oxidative stress, leading to cellular injury and death. Since CECs do not regenerate, they are particularly vulnerable to this type of damage, making it imperative to minimize exposure to oxidative stress during surgery [26].

Comparison of Endothelial Cell Loss Between "Direct-Chop" and "Stop-and-Chop"

The comparison of endothelial cell loss between the "direct-chop" and "stop-and-chop" techniques in phacoemulsification surgery reveals significant differences in outcomes regarding corneal endothelial health and surgical efficiency. Both techniques are widely used in cataract surgery, but their impact on endothelial cell preservation varies considerably [27]. The direct-chop technique is known for its efficiency in minimizing ultrasonic energy usage during the procedure. Studies consistently show that this method results in lower endothelial cell loss than the stop-and-chop approach. Research indicates that patients undergoing the direct-chop technique experience significantly less endothelial cell loss within the first few months post-surgery. This is particularly beneficial for maintaining corneal health, as excessive endothelial cell loss can lead to complications such as corneal edema and decreased visual acuity [27]. In contrast, while effective in emulsifying the lens, the stop-and-chop technique requires more ultrasonic energy and longer operative times. This increased energy usage can contribute to greater endothelial cell loss. Some studies have reported that the stop-and-chop method may not be as favorable for preserving endothelial cell density (ECD), especially in cases involving denser cataracts. The longer duration of surgery and the potential for more mechanical trauma during lens manipulation are contributing factors to the increased risk of endothelial damage associated with this technique [28]. Comparative studies further support these findings. A randomized controlled trial that examined both techniques found that the direct-chop method consistently resulted in lower cumulative dissipated energy (CDE) and less endothelial cell loss across various cataract densities. This suggests that the direct-chop approach is more protective of CECs. Additionally, another study indicated that the direct-chop technique is associated with reduced surgical time and less intraoperative manipulation, contributing to better preservation of endothelial cells [29].

Factors Influencing Endothelial Cell Outcomes

Several critical factors influence the health of CECs following phacoemulsification surgery. Understanding these factors is essential for optimizing surgical outcomes and preserving corneal integrity [30]. One of the most significant factors affecting endothelial cell outcomes is the surgeon's experience. Experienced surgeons tend to have refined techniques and better decision-making skills, which can lead to more efficient surgeries with reduced complications [30]. Their familiarity with different phacoemulsification methods allows them to select the most appropriate technique for each case, minimizing unnecessary trauma to the cornea and optimizing energy use. Studies have shown that surgeon experience correlates with lower rates of endothelial cell loss, highlighting the importance of skill and training in achieving favorable postoperative results [30]. The amount of ultrasound energy applied during phacoemulsification plays a crucial role in the health of the corneal endothelium. Higher levels of ultrasound energy are associated with increased endothelial cell damage, as excessive energy can lead to thermal injury and mechanical trauma. Techniques that minimize energy usage, such as the direct-chop method, are particularly beneficial for preserving endothelial cells. By effectively emulsifying the lens with less energy, these techniques help reduce the CDE, which is directly linked to endothelial cell loss. Consequently, careful management of ultrasound energy is vital for protecting corneal health during cataract surgery [31]. The stability of the anterior chamber during surgery is another critical factor influencing endothelial cell outcomes. A stable anterior chamber helps maintain the integrity of the cornea and reduces the risk of mechanical trauma to the endothelium. Techniques that ensure optimal anterior chamber depth and stability, such as using viscoelastic substances, can protect the corneal endothelium from damage during lens manipulation and emulsification. In contrast, fluctuations in anterior chamber pressure or instability can lead to increased contact between lens fragments and the endothelium, resulting in greater cell loss. Therefore, maintaining anterior chamber stability is essential for minimizing endothelial cell injury during phacoemulsification [32].

Effects on visual acuity

Definition and Importance of Visual Acuity in Cataract Surgery Outcomes

Visual acuity is a fundamental measure of visual function that quantifies the clarity or sharpness of vision. It is typically assessed using the Snellen eye chart, with results expressed in a fraction format, such as 6/6 vision, which is considered normal. In this context, the numerator indicates the distance from which the test is conducted (6 meters). At the same time, the denominator represents the distance at which a person with normal vision can read the same line [33]. For instance, 6/60 vision indicates significant visual impairment, as the individual can only see at 6 meters, which is what a person with normal vision can see at 60 meters. This metric is crucial in evaluating the effectiveness of various ophthalmic procedures, particularly cataract surgery [33]. In cataract surgery, visual acuity is a primary outcome measure, reflecting the surgery's success in restoring clear vision. The primary goal of phacoemulsification cataract surgery is to remove the opacified natural lens and replace it with an artificial IOL, thereby improving visual clarity [34]. Numerous studies have documented the effectiveness of cataract surgery in enhancing visual acuity. For instance, a meta-analysis revealed a mean improvement of 0.33 logMAR (approximately three lines on the Snellen chart) post-surgery. Additionally, population-based studies, such as one conducted in Hong Kong, indicated that 36.6% of patients achieved a presenting visual acuity of 6/18 or better in both eyes following the procedure [35]. However, the degree of visual acuity improvement can be influenced by various factors. The presence of ocular comorbidities, such as diabetic retinopathy, glaucoma, and age-related macular degeneration, can significantly limit the potential for visual enhancement [36]. Furthermore, patients with higher preoperative visual acuity may experience less dramatic improvements due to a ceiling effect, with limited room for enhancement. Refractive errors, both spherical and cylindrical, can also impede visual outcomes; research indicates that each diopter of refractive error may reduce the expected improvement in visual acuity. Surgical complications, such as vitreous loss or iris trauma, can also adversely affect the final visual results [37].

Impact of "Direct-Chop" on Postoperative Visual Acuity

The direct-chop technique in phacoemulsification has significantly impacted postoperative visual acuity across various studies. One notable study involving 115 eyes found that the proportion of patients with normal to mild visual impairment increased dramatically from 6.9% preoperatively to 84.3% by day 30 post-surgery. This substantial improvement underscores the effectiveness of the direct-chop technique in enhancing visual outcomes for cataract surgery patients [38]. Quantitative outcomes further validate the efficacy of the direct-chop method. In another study, patients experienced a mean best-corrected visual acuity (BCVA) improvement from 0.26 preoperatively to 0.82 at three months postoperatively, with statistically significant results (p<0.001). These findings suggest that the direct-chop technique effectively improves visual acuity and offers a reliable approach for cataract surgery [39]. Additionally, the direct-chop technique is associated with reduced ultrasound time and lower CDE, crucial for preserving corneal endothelial health. This efficiency in minimizing energy use helps reduce trauma to the ocular structures, thereby contributing to better postoperative visual outcomes. Protecting the corneal endothelium is vital for maintaining clear vision, and the direct-chop technique's ability to achieve this is a significant advantage [40]. Moreover, the safety profile of the direct-chop technique is favorable, with minimal complications reported in various studies. Intraoperative issues such as zonular dialysis were infrequent and not linked to significant adverse outcomes. This low complication rate reinforces the technique's reliability and effectiveness in achieving optimal visual acuity [41].

Impact of "Stop-and-Chop" on Postoperative Visual Acuity

The "stop-and-chop" technique in phacoemulsification cataract surgery has positively impacted postoperative visual acuity. This method is particularly effective for managing nuclear cataracts while minimizing complications and preserving CECs. Research shows that visual acuity significantly improves after cataract surgery using the stop-and-chop technique, with the mean BCVA typically increasing from a preoperative level of approximately 0.32 (logMAR) to about 0.65 postoperatively. This reflects a mean improvement of 0.33 (95% CI: 0.31-0.35), underscoring the technique's effectiveness in enhancing visual outcomes [42]. Studies have found that the stop-and-chop technique is as effective as the phaco-chop technique regarding visual acuity outcomes compared to other methods. Both techniques produce similar results regarding operative ultrasound power and overall surgical efficacy. This comparability allows surgeons to choose either method based on their preference and the patient's circumstances without compromising visual acuity results [39]. However, patient-specific factors can influence the degree of visual acuity improvement achieved with the stop-and-chop technique. For instance, diabetic retinopathy can reduce visual acuity improvement by approximately 0.095 logMAR, while glaucoma can decrease it by about 0.123 logMAR. These conditions can complicate the surgical process and affect overall visual outcomes, highlighting the importance of thorough preoperative assessments [43]. In addition to these factors, the stop-and-chop technique's efficiency in fragmenting the nucleus contributes to reduced ultrasound energy usage, which is crucial for maintaining corneal health. Lower endothelial cell loss is essential for optimal visual outcomes post-surgery, as a healthy corneal endothelium is vital for clear vision. In summary, while the stop-and-chop technique effectively improves postoperative visual acuity, careful consideration of patient-specific factors is necessary to maximize the benefits of this surgical approach [44].

Comparison of Visual Acuity Outcomes Between the Two Techniques

The comparison of visual acuity outcomes between the direct-chop and stop-and-chop techniques in phacoemulsification cataract surgery reveals that both methods are effective. However, they exhibit differences based on various parameters. Studies indicate that both techniques achieve comparable BCVA outcomes postoperatively. For instance, research shows that all patients in both groups achieved a BCVA of 6/6 after three weeks. This suggests that both methods are equally effective in improving visual acuity in patients with uncomplicated senile cataracts [45]. Regarding surgical efficiency, the direct-chop technique often shows a slight advantage over stop-and-chop. The mean effective phacoemulsification time is typically lower in the direct-chop group, which indicates a potential benefit in reducing the duration of the surgery. This increased efficiency is particularly valuable in minimizing the exposure of CECs to ultrasound energy, a crucial factor for postoperative recovery [39]. When considering endothelial cell loss, the direct-chop technique is associated with less CDE and lower endothelial cell loss compared to stop-and-chop, especially in cases with denser cataracts. Reduced energy usage helps preserve corneal health, which may also positively impact visual outcomes, as a healthier corneal endothelium contributes to overall visual quality [46]. Both techniques generally exhibit similar rates of postoperative complications. However, due to its efficient nuclear fragmentation, the direct-chop technique may lead to fewer intraoperative challenges. This can result in a smoother surgical experience and quicker patient recovery times. While both direct-chop and stop-and-chop techniques effectively achieve good visual acuity outcomes, the direct-chop technique may offer surgical efficiency and corneal endothelial preservation advantages, which are crucial for maintaining long-term ocular health [47].

Topical anesthesia in phacoemulsification

Overview of Anesthesia Options in Cataract Surgery

Cataract surgery is a common procedure, and the choice of anesthesia plays a crucial role in ensuring patient comfort and safety. Various anesthesia options are available, each with its advantages and considerations. Topical anesthesia is the most widely used technique in cataract surgery. This method involves applying numbing eye drops directly to the eye, providing adequate analgesia without requiring injections. Patients remain awake and conscious during the procedure, allowing them to follow instructions and focus on a microscope light. One of the significant benefits of topical anesthesia is its lower risk profile compared to injected methods. It minimizes complications associated with needles, such as hemorrhage or globe perforation. Additionally, intracameral lidocaine can enhance topical anesthesia, which is injected into the eye at the start of the surgery to further reduce discomfort [48]. Injected anesthesia, such as a sub-Tenon's block or retrobulbar block, provides a deeper level of anesthesia than topical methods. This technique involves injecting anesthetic under the conjunctiva and Tenon's capsule, which not only numbs the eye but also helps immobilize it during the procedure. While effective, injected anesthesia carries a slightly higher risk of complications, including retrobulbar hemorrhage or damage to the optic nerve. It is generally used in cases where patients require more extensive pain control or have difficulty cooperating during surgery [49]. In many cases, intravenous (IV) sedation is used in conjunction with either topical or injected anesthesia to enhance patient comfort. Sedation helps patients feel calm and drowsy, reducing anxiety and discomfort during the procedure. While patients remain conscious and able to cooperate, sedation can induce a degree of amnesia, meaning they may not remember parts of the surgery, which can be beneficial for those who experience anxiety [50]. General anesthesia is rarely necessary for routine cataract surgery. It is typically reserved for patients who cannot cooperate or remain under local anesthesia. While general anesthesia can provide a completely pain-free experience, it carries higher risks compared to local techniques, including respiratory complications and longer recovery times [51].

Benefits of Topical Anesthesia

Topical anesthesia offers several significant benefits, especially in medical procedures such as ophthalmic surgeries. One of the primary advantages is its ability to effectively manage pain and discomfort during procedures. In ophthalmology, for instance, topical anesthetics can significantly reduce pain associated with cataract surgery compared to traditional methods like injections. This results in higher patient satisfaction and a more relaxed surgical environment [52]. Another important benefit of topical anesthesia is the minimized risk of complications. By eliminating the need for injections, the likelihood of issues such as hematoma formation and nerve damage is greatly reduced. This is particularly crucial in delicate areas like the eye, where precision is essential. Additionally, patients often experience quicker recovery times with topical anesthesia. The reduced postoperative pain and discomfort can contribute to faster healing and a smoother recovery process, allowing patients to return to their daily activities sooner [53]. Topical anesthetics also avoid the pain associated with needle injections, which can be anxiety-inducing for many patients. This is especially advantageous for pediatric populations or individuals who fear needles. The versatility and ease of use of topical anesthetics further enhance their appeal. Available in various forms, such as gels, sprays, and creams, they can be easily applied to the affected area, providing flexibility in administration based on the specific procedure and patient needs [54]. Moreover, the use of topical anesthesia can lead to improved cosmetic outcomes. Since these agents do not distort tissue, they can result in better aesthetic results post-procedure, which is particularly relevant in surgeries where appearance is a significant concern. Finally, topical anesthesia supports the increasing trend of outpatient procedures by allowing for effective pain management without the need for more invasive anesthetic techniques. This facilitates quicker patient turnover in clinical settings, making it a practical choice for healthcare providers and patients [55].

Challenges and Considerations With Topical Anesthesia in Phacoemulsification

Topical anesthesia in phacoemulsification cataract surgery offers several advantages, such as reduced pain associated with injections and a lower risk of complications. However, it also presents challenges and considerations that can impact patient comfort and surgical outcomes [8]. One of the primary challenges with topical anesthesia is effective pain management. While many patients tolerate the procedure well, some report discomfort, particularly during specific surgical steps such as hydrodissection and hydroaspiration. This discomfort can vary based on individual patient characteristics, including age and the axial length of the eye [56]. Younger patients and those with longer eyes often experience more pain, highlighting the need for tailored pain management strategies. Although topical anesthesia is generally effective, it may not always provide adequate analgesia, especially in more complex surgeries or with denser cataracts. Research indicates that supplementing topical anesthesia with intracameral lidocaine can significantly reduce intraoperative pain levels, although the clinical significance of this reduction is still debated [56]. Another consideration is the variability in patient experiences with topical anesthesia. Individual factors such as pain tolerance, anxiety levels, and previous surgical experiences can influence how patients perceive discomfort during the procedure. This variability complicates the assessment of anesthesia effectiveness and overall patient satisfaction. Surgeons must be aware of these differences and be prepared to adjust their approach based on individual patient needs [57]. The skill and experience of the surgeon play a crucial role in maximizing the effectiveness of topical anesthesia. More experienced surgeons may achieve better outcomes, with patients reporting less pain during the procedure. Conversely, less experienced surgeons may face challenges in managing discomfort effectively, particularly in complex cases. Therefore, a thorough preoperative assessment is essential to identify patients at higher risk for pain during surgery. Factors such as previous ocular surgeries, anxiety, and specific anatomical considerations should be evaluated to tailor the anesthesia approach appropriately [58]. Finally, proper surgical setup can significantly impact patient comfort. For instance, maintaining an appropriate infusion height is vital to prevent excessive distention of the anterior chamber, a common source of discomfort during surgery. By optimizing these factors, surgeons can enhance the overall experience for patients undergoing cataract surgery with topical anesthesia [59].

Comparison of Patient Outcomes and Comfort Between Techniques Under Topical Anesthesia

Comparing patient outcomes and comfort between phacoemulsification techniques performed under topical anesthesia offers valuable insights into different approaches' effectiveness and overall experience. Topical anesthesia, which involves applying anesthetic drops directly to the eye, has become popular due to its minimal invasiveness and lower risk of complications compared to traditional injection methods [60]. In terms of patient comfort and pain perception, studies indicate that while topical anesthesia alone is effective, supplementing it with intracameral lidocaine can significantly improve pain management. Research shows that patients receiving intracameral lidocaine report lower intraoperative pain scores compared to those using topical anesthesia alone. Despite this, pain levels during surgery are generally low for most patients, even without the supplement, falling within a minimal discomfort range. This suggests both methods provide adequate analgesia during phacoemulsification, contributing to a generally positive patient experience [61]. Patient satisfaction is another crucial aspect of this comparison. Many patients undergoing phacoemulsification with topical anesthesia report high levels of satisfaction, largely due to the absence of injection-related complications and a reduced risk of ocular injury. The comfort associated with avoiding needles, combined with effective pain management, enhances the overall surgical experience for patients [62]. Regarding surgical outcomes, phacoemulsification performed under topical anesthesia demonstrates favorable results. Patients typically experience significant improvements in visual acuity postoperatively, with many achieving good BCVA within a month after surgery [35]. Importantly, the choice of anesthesia does not appear to increase the risk of intraoperative complications. Studies have shown that both topical anesthesia alone and its supplementation with intracameral lidocaine result in similar rates of complications during surgery, reinforcing the safety and efficacy of topical anesthesia approaches [35].

Clinical studies and data analysis

Recent clinical studies have provided valuable insights into the efficacy and safety of the direct-chop and stop-and-chop techniques in phacoemulsification. A notable randomized controlled trial compared three techniques, stop-and-chop, direct-chop, and terminal-chop, involving 307 eyes. In this study, 102 eyes were assigned to the stop-and-chop group, 103 to the direct-chop group, and 102 to the terminal-chop group. Key parameters evaluated included central corneal thickness (CCT), ECD, CDE, and BCVA. The results indicated that while both direct-chop and stop-and-chop techniques were effective, the terminal-chop technique showed statistically significant advantages in minimizing endothelial cell loss and CDE, particularly in denser cataracts classified as NS II and NS IV. However, no significant differences were observed between the direct-chop and stop-and-chop techniques across various cataract grades, suggesting that both methods are comparably effective regarding safety and visual outcomes when performed by experienced surgeons [63]. Data analysis from these studies revealed that both the direct-chop and stop-and-chop techniques reduced endothelial cell count postoperatively. Specifically, endothelial cell loss was generally minimal with the direct-chop technique compared to stop-and-chop, with some studies highlighting the benefits of direct-chop in reducing ultrasound time and energy usage. Both techniques yielded favorable outcomes regarding visual acuity, with no significant differences in BCVA between the two methods. However, some studies suggested that the direct-chop technique might offer slightly better visual outcomes due to its efficiency in lens fragmentation and reduced energy application, which is critical for preserving corneal health [63]. The statistical analysis of the clinical studies revealed varying degrees of significance regarding the outcomes of the different techniques. For instance, the study comparing the three techniques found significant differences in postoperative CCT and endothelial cell loss, particularly favoring the terminal-chop technique in specific cataract grades, with p-values of 0.0001 and 0.005, respectively. In contrast, direct-chop and stop-and-chop comparisons showed no statistically significant differences, indicating that both techniques are effective with similar safety profiles. The statistical methods, including descriptive statistics and comparative analysis, ensured the findings were robust and reliable across the evaluated parameters [63].

Limitations of the current research

Current research on phacoemulsification techniques, particularly the direct-chop and stop-and-chop methods, presents several limitations that impact the interpretation and application of findings. One primary limitation is the variability in surgical skills and experience among practitioners. The effectiveness of these techniques can significantly depend on the surgeon's proficiency, introducing variability in outcomes. Many studies do not account for this variability, making generalizing results across different clinical settings challenging. Additionally, the lack of standardized protocols for measuring outcomes such as endothelial cell loss and visual acuity can lead to inconsistencies in data reporting and analysis across studies [64]. Another limitation is the sample size and demographic diversity in clinical trials. Many studies focus on a limited population that may not accurately represent the broader patient demographic. Variations in age, pre-existing ocular conditions, and cataract density among participants can influence surgical outcomes, yet these factors are often inadequately controlled. Moreover, the follow-up duration in many studies is relatively short, which may not capture long-term effects on endothelial cell health and visual acuity [65]. The reliance on specific technologies and equipment also poses limitations. The costs associated with advanced phacoemulsification devices and disposable instruments can restrict their use in underdeveloped regions, potentially skewing research outcomes towards those observed in more affluent healthcare settings. This economic barrier can lead to a lack of comprehensive data on the effectiveness of these techniques in diverse healthcare environments [66]. Finally, while many studies emphasize the advantages of newer techniques like direct-chop, there is often insufficient comparative analysis with traditional methods. This can create a bias towards newer techniques without adequately assessing their relative efficacy and safety in various clinical scenarios. To address these issues, more extensive, multicenter studies with diverse populations and standardized outcome measures are necessary to better understand the limitations and benefits of different phacoemulsification techniques [67].

## Conclusions

The "direct-chop" and "stop-and-chop" techniques in phacoemulsification each offer distinct advantages and challenges, particularly regarding the preservation of CECs and the maintenance of visual acuity under topical anesthesia. The "direct-chop" technique, with its efficiency in reducing phacoemulsification time and energy, shows promise in better preserving endothelial cells, which is crucial for maintaining corneal transparency and overall eye health. Conversely, the "stop-and-chop" technique provides a balance of control and safety, making it a reliable option for surgeons, especially those less experienced. Both techniques, when performed skillfully, can result in excellent visual outcomes for patients. This review underscores the importance of individualized surgical planning and technique selection based on the specific needs and conditions of the patient. Future research should continue to explore the nuances of these techniques, with an emphasis on long-term outcomes and patient satisfaction, to further refine cataract surgery practices and improve overall patient care.
